# Efficacy of methotrexate, doxorubicin, and cisplatin for osteosarcoma: Study protocol for a systematic review of randomized controlled trial

**DOI:** 10.1097/MD.0000000000014442

**Published:** 2019-02-08

**Authors:** Qing-xi Tang, Lu-Chuan Wang, Yu Wang, Hong-da Gao, Zhi-ling Hou

**Affiliations:** a First Ward of Orthopedis Department; b First Ward of Orthopedis Department, Jiamusi Hospital of Traditional Chinese Medicine; c First Ward of Orthopedis Department, Jiamusi Central Hospital; d Department of Emergency Surgery, First Affiliated Hospital of Jiamusi University, Jiamusi, China.

**Keywords:** doxorubicin and cisplatin, efficacy, methotrexate, osteosarcoma, randomized controlled trial, safety

## Abstract

**Background:**

This systematic review will address the efficacy and safety of methotrexate, doxorubicin, and cisplatin (MAP) for the treatment of patients with osteosarcoma.

**Methods:**

We will retrieve the studies from the following 9 electronic databases: Cochrane Central Register of Controlled Trials, EMBASE, MEDLINE, the Cumulative Index to Nursing and Allied Health Literature, the Allied and Complementary Medicine Database, and Chinese Biomedical Literature Database, China National Knowledge Infrastructure, VIP Information, and Wanfang Data. Two independent researchers will screen and select the relevant papers for eligibility after the search strategies have been conducted. All articles up to the present in any language, region will be considered in this study. A systematic review and data synthesis will be performed of randomized controlled trials of MAP for the treatment of patients with osteosarcoma. The primary outcome includes event-free survival. The secondary outcomes consist of overall survival, quality of life, and toxicity. In addition, 2 independent researchers will extract data, and will assess the quality of included studies by using Cochrane risk of bias tool. Results data will be pooled and meta-analysis will be conducted if >2 eligible studies will be included.

**Results:**

This systematic review will evaluate the efficacy and safety of MAP for the treatment of patients with osteosarcoma.

**Conclusion:**

The findings of this study will summarize the up-to-date evidence of MAP for osteosarcoma, and may provide the guidance for the clinical practice, as well as the health policy maker.

**PROSPERO registration number:**

PROSPERO CRD42018120004.

## 1 Introduction

Osteosarcoma is one of the most frequent primary sarcoma of bone among young population.^[1–3]^ Although it has been reported that its overall prevalence and incidence is rare with only 3 persons per million suffer from this condition annually, its treatment is very tough and tricky.^[4–7]^ Current treatment strategy usually consists of several weeks of chemotherapy before the surgery, then following by the surgery, and also several weeks of chemotherapy after the surgery.^[8–11]^ However, the overall outcome results were disappointed and unsatisfied during the past decades.^[12–14]^

Present standard chemotherapy for the treatment of this disorder is the combination of methotrexate, doxorubicin, and cisplatin, also known as MAP.^[15–19]^ However, its efficacy is still inconclusive. Furthermore, no systematic review and meta-analysis has been conducted to assess the efficacy and safety of MAP for osteosarcoma. Therefore, in the present protocol of systematic review, we will aim to evaluate the efficacy and safety of MAP for the treatment of osteosarcoma.

## 2 Methods

### 2.1 Study registration

This protocol has been registered with PROSPERO (CRD42018120004), and has been reported in according with the Preferred Reporting Items for Systematic Reviews and Meta-Analysis (PRISMA) Protocol statement guidelines.^[20]^

### 2.2 Study selection criteria

#### 2.2.1 Study types

Only randomized controlled trials (RCTs) of MAP alone for osteosarcoma will be included. All other study types will not be included, such as reviews, non-clinical trials, case reports, letters, and so on.

#### 2.2.2 Interventions

Study reporting results of interventions involving MAP alone for osteosarcoma only will be included. The combinations of MAP with other interventions for osteosarcoma treatment will not be included. The control treatment will include any types of interventions, except the MAP.

#### 2.2.3 Population

Patients with osteosarcoma, regardless race, sex, and age will be considered to include in this study.

#### 2.2.4 Outcomes

The primary outcome is event-free survival. It is defined as the time from random assignment until a first event or censoring at last contact. The secondary outcomes include overall survival (defined as time from random assignment until death resulting from any cause or last contact), quality of life (as measured by any measurement tools, such as 36-Item Short Form Health Survey), and toxicity (any short- or long-term toxicities).

#### 2.2.5 Search strategy

Nine databases of Cochrane Central Register of Controlled Trials (CENTRAL, present), Embase (1980 to present), MEDLINE (1946 to present), the Cumulative Index to Nursing and Allied Health Literature (CINAHL, 1982 to present), the Allied and Complementary Medicine Database (AMED, 1985 to present), and 4 Chinese database Chinese Biomedical Literature Database (CBM, 1980 to present), China National Knowledge Infrastructure (which includes the database China Academic Journals) (CNKI, 1980 to present), VIP Information (VIP, 1980 to present), and Wanfang Data (WANFANG, 1980 to present) will be searched for the relevant trials. The sample search strategy for CENTRAL has been developed according to the consolation results of a subject-specific librarian (Table 1) and will be applied to other electronic databases. In addition, clinical registration website, and reference lists of potentially related studies will also be retrieved to avoid missing any potential eligible studies.

**Table 1:**
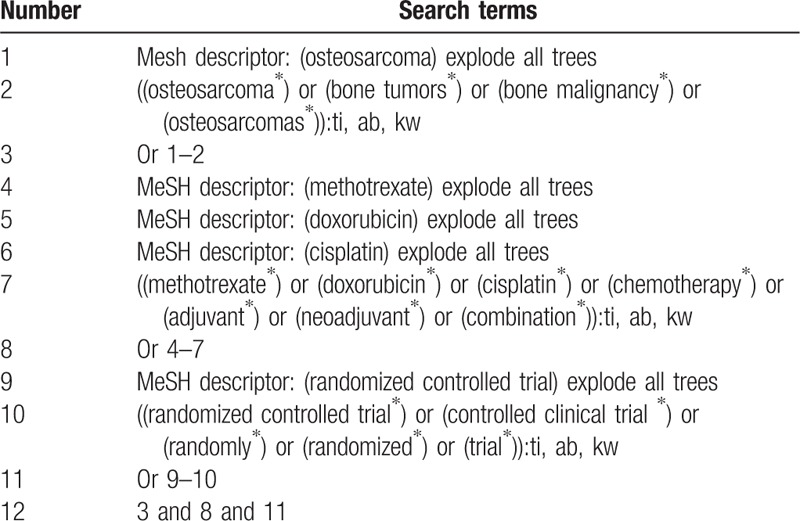
Search strategy applied in CENTRAL database.

#### 2.2.6 Study selection

**Figure 1:**
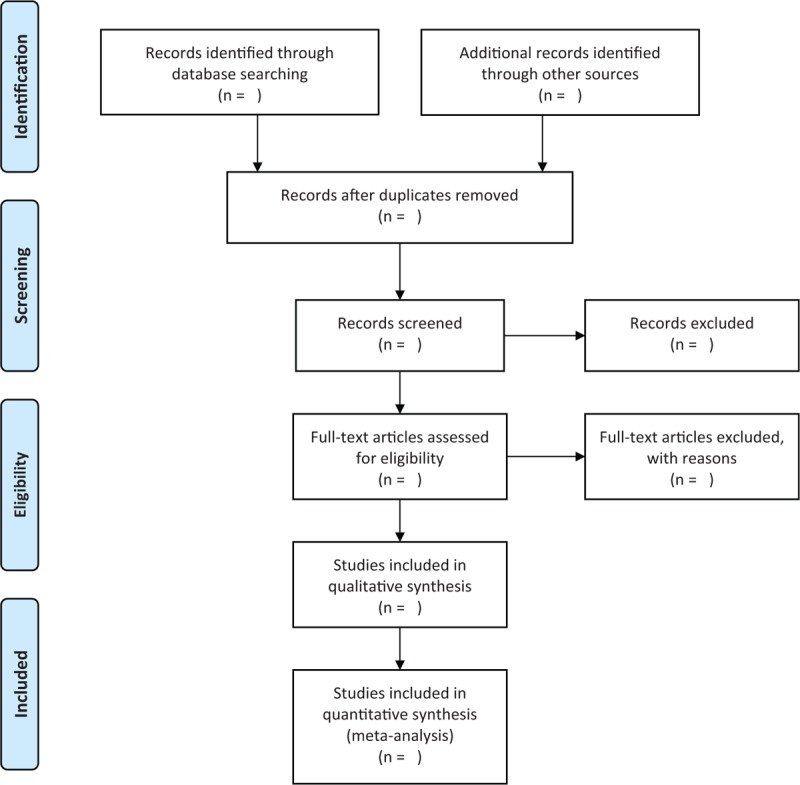
Flowchart of study selection.

Two independent researchers will conduct the study selection by screening titles and abstracts initially. Then, full texts of potential studies will be further reviewed by reading full papers. All procedures of study selection will be based on the PRISMA flow chart. Any differences between 2 researchers will be solved by a third researcher involved by discussion. The flowchart of study selection is showed in Fig. 1.

### 2.3 Data extraction and management

Two independent researchers will extract all related data from the available included studies. The extracted information will consist of basic information (authors, published year, location, age, sex, funding, and setting), study methods (study design, sample size, details of randomization, allocation, and blinding), interventions (details of treatments from both groups, such as dosage, treatment duration), and outcomes (including primary, secondary outcomes, and any other measurements). The divergences between 2 researchers will be resolved by a third researcher through consultation. Data obtained from the results of exaction will be entered into excel. Outcome results will be pooled into RevMan software 5.3 (The Cochrane Collaboration, London, UK) for meta-analysis.

### 2.4 Risk of bias assessment

Risk of bias for each included study will be used with the Cochrane Risk of Bias Tool. This tool includes 7 items, and each item is assessed by categorizing with low risk of bias, unclear risk of bias, and high risk of bias. Two independent researchers will evaluate the risk of bias for each included study. Any divisions will also be settled down by a third author invited through discussion.

### 2.5 Measurement of treatment effect

The continuous outcome data will be presented as mean difference (MD) or standardized mean difference (SMD) with 95% confidence intervals (CIs), while the dichotomous outcome data will be presented as risk ratio (RR) with 95% CIs.

### 2.6 Unit of analysis

If the cross-over studies will be included in this study, we will only evaluate the first period of study data.

### 2.7 Missing data

If the included study has missing, insufficient or unclear data, we will contact the original corresponding authors to inquire those data. If those data will not be required, we will just pool and analyze the present available data. In addition, it will also be discussed in the manuscript.

### 2.8 Heterogeneity assessment

In this study, we will utilize the *I*^2^ and chi-squared tests to assess the heterogeneity. The value of *I*^2^ is <50%, heterogeneity is considered as reasonable, while the value of *I*^2^ is >50%, heterogeneity is defined as significant.

### 2.9 Data synthesis

If reasonable heterogeneity is identified, fixed-effect model will be used to pool the data, and the meta-analysis will also be conducted. If the heterogeneity is significant, the random-effect model will be utilized to pool the data. In addition, the subgroup analysis will be performed to identify any potential reasons that may cause heterogeneity. If the heterogeneity is still substantial after the subgroup analysis, then data will not be pooled, and meta-analysis will not be conducted. Instead, a narrative summary will be elaborated.

### 2.10 Subgroup analysis

We will conduct the subgroup analysis if significant heterogeneity will be detected according to the differences of treatments, controls, as well as the outcome assessment tools.

### 2.11 Sensitivity analysis

If the data can be pooled, and meta-analysis can be conducted, then sensitivity analysis will be performed to identify the robustness of pooled results, methodological quality, and missing data of all included studies.

### 2.12 Publication bias

If >10 included studies are available, funnel plot will be used to identify the possible publication bias.^[21]^ Additionally, Egg regression and Begger tests will be utilized to detect the funnel plot asymmetry.^[22]^

## 3 Discussion

The protocol of this systematic review will utilize rigorous methodology to detect and examine studies reporting the outcomes of MAP for osteosarcoma. No systematic review has previously addressed this issue, although lots of published reviews have explored the issue of MAP for osteosarcoma. Although the potential risk of bias of the included studies may limit the analysis, as well as the power of the results in this systematic review, the data pooled results will provide a better understanding of the efficacy of MAP for patients with osteosarcoma.

This review will provide the first rigorous summary evidence of MAP for osteosarcoma across all published randomized controlled trials. The findings of this systematic review will inform our understanding of the value of MAP in treating osteosarcoma outcomes. This evidence may also provide helpful evidence for clinical practice and health policy-makers for the treatment of osteosarcoma.

## Author contributions

**Conceptualization:** Qing-xi Tang, Yu Wang, Zhi-ling Hou.

**Data curation:** Qing-xi Tang, Lu-Chuan Wang, Yu Wang, Hong-da Gao, Zhi-ling Hou.

**Formal analysis:** Lu-Chuan Wang, Hong-da Gao, Zhi-ling Hou.

**Funding acquisition:** Zhi-ling Hou.

**Methodology:** Qing-xi Tang, Yu Wang, Zhi-ling Hou.

**Project administration:** Zhi-ling Hou.

**Resources:** Qing-xi Tang, Lu-Chuan Wang, Yu Wang, Hong-da Gao, Zhi-ling Hou.

**Software:** Qing-xi Tang, Yu Wang, Hong-da Gao, Zhi-ling Hou.

**Supervision:** Zhi-ling Hou.

**Validation:** Qing-xi Tang, Lu-Chuan Wang, Hong-da Gao.

**Visualization:** Qing-xi Tang, Lu-Chuan Wang, Hong-da Gao.

**Writing – original draft:** Qing-xi Tang, Lu-Chuan Wang, Zhi-ling Hou.

**Writing – review & editing:** Qing-xi Tang, Yu Wang, Hong-da Gao, Zhi-ling Hou.

## References

[1] Zhang Y Yang J Zhao N Progress in the chemotherapeutic treatment of osteosarcoma. Oncol Lett 2018;16:6228–37. 30405759 10.3892/ol.2018.9434 PMC6202490

[2] Wang X Liu Z Systematic meta-analysis of genetic variants associated with osteosarcoma susceptibility. Medicine (Baltimore) 2018;97:e12525. 30235774 10.1097/MD.0000000000012525 PMC6160068

[3] Bacci G Briccoli A Ferrari S Neoadjuvant chemotherapy for osteosarcoma of the extremities with synchronous lung metastases: treatment with cisplatin, adriamycin and high dose of methotrexate and ifosfamide. Oncol Rep 2000;7:339–46. 10671683 10.3892/or.7.2.339

[4] Lan M Zhu XP Cao ZY Extracellular vesicles-mediated signaling in the osteosarcoma microenvironment: roles and potential therapeutic targets. J Bone Oncol 2018;12:101–4. 30155405 10.1016/j.jbo.2018.07.010 PMC6111053

[5] Hameed M Mandelker D Tumor syndromes predisposing to osteosarcoma. Adv Anat Pathol 2018;25:217–22. 29668499 10.1097/PAP.0000000000000190 PMC6688172

[6] Yang Y Han L He Z Advances in limb salvage treatment of osteosarcoma. J Bone Oncol 2017;10:36–40. 29296558 10.1016/j.jbo.2017.11.005 PMC5739147

[7] Fu Y Lan T Cai H Meta-analysis of serum lactate dehydrogenase and prognosis for osteosarcoma. Medicine (Baltimore) 2018;97:e0741. 29742740 10.1097/MD.0000000000010741 PMC5959391

[8] Gorlick R Bielack S Teot L Pizzo P Poplack D Osteosarcoma: biology, diagnosis, treatment, and remaining challenges. Principles and Practice of Pediatric Oncology 6th ed. Philadelphia, PA: Lippincott Williams and Wilkins; 2011 1015–44.

[9] Kumar R Kumar M Malhotra K Primary osteosarcoma in the elderly revisited: current concepts in diagnosis and treatment. Curr Oncol Rep 2018;20:13. 29492676 10.1007/s11912-018-0658-1

[10] Harrison DJ Geller DS Gill JD Current and future therapeutic approaches for osteosarcoma. Expert Rev Anticancer Ther 2018;18:39–50. 29210294 10.1080/14737140.2018.1413939

[11] Biazzo A De Paolis M Multidisciplinary approach to osteosarcoma. Acta Orthop Belg 2016;82:690–8. 29182106

[12] Jimmy R Stern C Lisy K Effectiveness of mifamurtide in addition to standard chemotherapy for high-grade osteosarcoma: a systematic review. JBI Database System Rev Implement Rep 2017;15:2113–52. 10.11124/JBISRIR-2016-003105 28800058

[13] Mirabello L Troisi RJ Savage SA Osteosarcoma incidence and survival rates from 1973 to 2004: data from the Surveillance, Epidemiology, and End Results Program. Cancer 2009;115:1531–43. 19197972 10.1002/cncr.24121 PMC2813207

[14] Stiller CA Bielack SS Jundt G Bone tumours in European children and adolescents, 1978–1997: report from the Automated Childhood Cancer Information System project. Eur J Cancer 2006;42:2124–35. 16919776 10.1016/j.ejca.2006.05.015

[15] Meyers PA Schwartz CL Krailo MD Osteosarcoma: the addition of muramyl tripeptide to chemotherapy improves overall survival—a report from the Children's Oncology Group. J Clin Oncol 2008;26:633–8. 18235123 10.1200/JCO.2008.14.0095

[16] Smeland S Bruland OS Hjorth L Results of the Scandinavian Sarcoma Group XIV protocol for classical osteosarcoma: 63 patients with a minimum follow-up of 4 years. Acta Orthop 2011;82:211–6. 21434784 10.3109/17453674.2011.566141 PMC3235293

[17] Whelan J Patterson D Perisoglou M The role of interferons in the treatment of osteosarcoma. Pediatr Blood Cancer 2010;54:350–4. 19902521 10.1002/pbc.22136

[18] Kudawara I Aoki Y Ueda T Neoadjuvant and adjuvant chemotherapy with high-dose ifosfamide, doxorubicin, cisplatin and high-dose methotrexate in non-metastatic osteosarcoma of the extremities: a phase II trial in Japan. J Chemother 2013;25:41–8. 23433444 10.1179/1973947812Y.0000000055

[19] Bielack SS Smeland S Whelan JS Methotrexate, doxorubicin, and cisplatin (MAP) plus maintenance pegylated interferon alfa-2b versus MAP alone in patients with resectable high-grade osteosarcoma and good histologic response to preoperative MAP: first results of the EURAMOS-1 good response randomized controlled trial. J Clin Oncol 2015;33:2279–87. 26033801 10.1200/JCO.2014.60.0734 PMC4486345

[20] Shamseer L Moher D Clarke M PRISMA-P Group. Preferred reporting items for systematic review and meta-analysis protocols (PRISMA-P) 2015: elaboration and explanation. BMJ 2015;349:g7647. 10.1136/bmj.g7647 25555855

[21] Sutton AJ Duval SJ Tweedie RL Empirical assessment of effect of publication bias on meta-analyses. BMJ 2000;320:1574–7. 10845965 10.1136/bmj.320.7249.1574 PMC27401

[22] Egger M Davey Smith G Schneider M Bias in meta-analysis detected by a simple, graphical test. BMJ 1997;315:629–34. 9310563 10.1136/bmj.315.7109.629 PMC2127453

